# Peroxisome Proliferator–Activated Receptor-γ Mediates Bisphenol A Inhibition of FSH-Stimulated IGF-1, Aromatase, and Estradiol in Human Granulosa Cells

**DOI:** 10.1289/ehp.0901161

**Published:** 2009-10-22

**Authors:** Jakub Kwintkiewicz, Yoshihiro Nishi, Toshihiko Yanase, Linda C. Giudice

**Affiliations:** 1 Department of Obstetrics, Gynecology and Reproductive Sciences, University of California San Francisco, San Francisco, California, USA; 2 Department of Physiology, Kurume University School of Medicine, Kurume, Fukuoka, Japan; 3 Department of Medicine and Bioregulatory Science, Graduate School of Medical Sciences, Kyushu University, Fukuoka, Japan

**Keywords:** aromatase, bisphenol A, estradiol, FSH, granulosa, IGF-1, ovary, PPARγ

## Abstract

**Background:**

Bisphenol A (BPA), a chemical used as a plasticizer, is a potent endocrine disruptor that, even in low concentrations, disturbs normal development and functions of reproductive organs in different species.

**Objectives:**

We investigated whether BPA affects human ovarian granulosa cell function.

**Methods:**

We treated KGN granulosa cells and granulosa cells from subjects undergoing *in vitro* fertilization (IVF) with follicle-stimulating hormone (FSH), BPA, or BPA plus FSH in a dose- and time-dependent manner. We then evaluated expression of insulin-like growth factor 1 (IGF-1), aromatase, and transcription factors known to mediate aromatase induction by FSH [including steroidogenic factor-1 (SF-1), GATA4, cAMP response element binding protein-1 (CREB-1), and peroxisome proliferator–activated receptor-γ (PPARγ)], as well as 17β-estradiol (E_2_) secretion. KGN cells were transfected with a PPARγ-containing vector, followed by assessment of aromatase and IGF-I expression.

**Results:**

BPA reduced FSH-induced IGF-1 and aromatase expression and E_2_ secretion in a dose-dependent fashion. Similar effects on aromatase were observed in IVF granulosa cells. SF-1 and GATA4, but not CREB-1, were reduced after BPA treatment, although PPARγ, an inhibitor of aromatase, was significantly up-regulated by BPA in a dose-dependent manner, with simultaneous decrease of aromatase. Overexpression of PPARγ in KGN cells reduced FSH-stimulated aromatase and *IGF*-*1* mRNAs, with increasing concentrations of the transfected expression vector, mimicking BPA action. Also, BPA reduced granulosa cell DNA synthesis without changing DNA fragmentation, suggesting that BPA does not induce apoptosis.

**Conclusions:**

Overall, the data demonstrate that BPA induces PPARγ, which mediates down-regulation of FSH-stimulated IGF-1, SF-1, GATA4, aromatase, and E_2_ in human granulosa cells. These observations support a potential role of altered steroidogenesis and proliferation within the ovarian follicular compartment due to this endocrine disruptor.

Bisphenol A (BPA), a compound widely used in industry, is present in polycarbonate plastics, epoxy resins, metal cans and water pipes, beverage and baby formula bottles, eyeglass lenses, toys, sports equipment, dental sealants, medical equipment and tubing, and consumer electronics (Vandenberg et al. 2007), making human exposure very prevalent. BPA has been found in serum [0.2–20 ng/mL (0.1–10 μM)] and in tissues of men and women of different ages and ethnic groups, with the highest concentration exceeding 100 ng/g in human placenta (Schonfelder et al. 2002). Concern about BPA arose in recent years when several studies reported adverse effects of this chemical on endocrine systems, especially the reproductive system and the thyroid (Zoeller 2007), leading to its designation as an endocrine disruptor. BPA exposure at different developmental stages disrupts meiotic spindle assembly and early oogenesis (Hunt et al. 2003; Susiarjo et al. 2007; Susiarjo and Hunt 2008) and steroidogenesis (Akingbemi et al. 2004; Lee et al. 2006; Mlynarcíková et al. 2005; Song et al. 2002; Zhou et al. 2008). It also adversely affects the nervous system (Farabollini et al. 2002; Wetherill et al. 2007) and the immune system (Richter et al. 2007; Yoshino et al. 2004).

Dominant follicle selection, growth, and maturation, as well as ovulation, oocyte quality, and subsequent corpus luteum function, depend on sequential actions of gonadotropins and intraovarian regulators, including the insulin-like growth factor (IGF) system (Fortune 1994; Kwintkiewicz and Giudice 2009). Granulosa aromatase [cytochrome P450 (CYP) 19] expression, key in the biosynthesis of ovarian-derived 17β-estradiol (E_2_), is stimulated by pituitary-derived follicle-stimulating hormone (FSH) and intraovarian IGFs during the follicular phase of the menstrual cycle (Hillier et al. 1994). FSH-driven aromatase expression in the granulosa cell compartment of the preovulatory follicle is controlled by the ovary-specific proximal PII promoter (Bulun et al. 2003) that contains response elements for transcription factors that regulate aromatase expression. These include steroidogenic factor-1 (SF-1), LRH-1 (liver receptor homolog-1), cAMP response element binding protein (CREB), and GATA4 (Bouchard et al. 2005; Carlone and Richards 1997; Kwintkiewicz et al. 2007) with resulting activation of the protein kinase A and other signal transduction pathways (Hunzicker-Dunn and Maizels 2006). BPA disrupts gonadal steroidogenesis in the rodent (Akingbemi et al. 2004; Song et al. 2002; Zhou et al. 2008), farm animals (Mlynarcíková et al. 2005), and wildlife species (Lee et al. 2006), although it is not clear what the mechanisms are for its actions. Interestingly, it has been proposed that despite its low affinity to estrogen receptors (ERs) compared with endogenous estrogens, BPA may act as a selective estrogen receptor modulator by impairing activation of ERα in various tissues (Hall and Korach 2002; Kurosawa et al. 2002; Routledge et al. 2000; Vivacqua et al. 2003).

Herein, we present evidence that exposure of human granulosa cells to BPA affects FSH-induced aromatase and E_2_ secretion, as well as other downstream effectors of FSH action, including IGF-1 and the transcription factors SF-1 and GATA4. Furthermore, data suggest that BPA may contribute to aromatase down-regulation via induction of peroxisome proliferator–activated receptor-γ (PPARγ). Overall the data suggest that the ubiquitously present endocrine disruptor BPA may affect the steroidogenic capacity in human ovary and seriously affect processes governing normal reproductive physiology and reproductive potential.

## Materials and Methods

### Cells, reagents, and cell culture

The KGN ovarian granulosa-like tumor cell line, which expresses a functional FSH receptor, was established from a patient with an invasive ovarian granulosa cell carcinoma, as described previously (Nishi et al. 2001). Frozen stocks of this cell line were thawed, and cells were plated in 24-well plates or 100-mm plates (125,000 cells/well or 1,000,000 cells/plate, respectively) and cultured in Dulbecco’s modified Eagle’s medium (DMEM)/F12 (Invitrogen, Carlsbad, CA, USA) at 37°C in 5% CO_2_ with the addition of 10% fetal bovine serum (FBS; Hyclone, Logan, UT, USA) until 70% confluent. KGN cells were then placed in serum-free DMEM/F12 medium at 37°C for 48 hr before treatments were added.

Human luteinized granulosa cells were isolated from follicular aspirates after oocyte retrieval from consenting subjects (*n* = 4) at the University of California San Francisco (UCSF) Center for Reproductive Health, under a protocol approved by the UCSF Committee on Human Research. Subjects (32 ± 3.3 years of age, mean ± SD) had undergone superovulation with gonadotropins after down-regulation with a gonadotropin-releasing hormone agonist. In all cases, oocyte retrieval was performed for treatment of male factor infertility. Human luteinized granulosa cells in follicular aspirates underwent a series of washes with calcium/magnesium-free phosphate-buffered saline (PBS) and were then centrifuged at 200 × *g*. Separation from blood cells was achieved on a Ficoll gradient, after which cells were plated in 24-well plates (125,000 cells/well) and cultured in DMEM/F12 at 37°C in 5% CO_2_ with the addition of 10% FBS until 70% confluent. Cells were then incubated in serum-free DMEM/F12 at 37°C for 48 hr before treatments were added.

OPTI-MEM medium and other cell culture reagents were purchased from Invitrogen. Human FSH (containing < 2% luteinizing hormone and < 1% each of thyroid-stimulating hormone, growth hormone, prolactin, and adrenocorticotropic hormone), BPA, (purity > 99%), and 4-androstene-3,17-dione (purity > 99%) were purchased from Sigma-Aldrich (St. Louis, MO, USA).

### RNA isolation and real-time polymerase chain reaction (PCR)

Total RNA from granulosa cells was isolated using TRIzol reagent (Invitrogen) following the manufacturer’s instructions. For mRNA analysis by reverse transcriptase PCR (RT-PCR), 0.5–1 μg of total RNA was reverse-transcribed at 42°C for 60 min using 200 U/μL M-MLV (Moloney murine leukemia virus) reverse transcriptase (Promega, Madison, WI, USA) in the presence of oligo(dT)_12–18_ primer (0.04 μg/μg of total RNA), 1 mM dNTP mix, and RNaseOUT ribonuclease inhibitor (1 U/μL; Invitrogen). After transcription, samples were diluted to a final volume of 200 μL with diethyl pyrocarbonate (DEPC)-treated water. Aliquots (5 μL) of sample cDNA were combined with Brilliant SYBR Green IQPCR Mastermix (Stratagene, La Jolla, CA, USA), specific primers for human aromatase (*CYP19*), *GATA4*, *SF-1*, *CREB*, *PPAR*γ, or *L19*, and water to a final volume of 25 μL. Intron-spanning primers were used for amplification of *CYP19*: (forward 5′-tgcatggcaagctctcctca-3′ and reverse 5′-tttgcgcatgaccaagtcca-3′), *GATA4* (forward 5′-tggcctgtcatctcactacg-3′ and reverse 5′-aagaccaggctgttccaaga-3′), *SF*-*1* (forward 5′-ctcaagttcatcatcctctt-3′ and reverse 5′-aggtactccttggcctgcat-3′), *CREB* (forward 5′-ctagcactattgcccctgga-3′ and reverse 5′-tttcaagcactgccactctg-3′), *PPAR*γ (forward 5′-gagggccaaggcttcatga-3′ and reverse 5′-aggctttcgcaggctctttag-3′), and *L19* (forward 5′-gcagataatgggaggagcc-3′ and reverse 5′-gcccatctttgatgagcttc-3′). Real-time quantification of the PCR product in each cycle was carried out using an Mx3005 QPCR system (Stratagene) with the following cycling conditions: preincubation at 95°C for 15 min, followed by 40 cycles of denaturation at 95°C for 15 sec, annealing at 58°C for 45 sec, and extension at 72°C for 60 sec. The melting peak of each sample was routinely determined by melting curve analysis to ascertain that only the expected products had been generated. The minimal number of cycles sufficient to produce detectable levels of fluorescence (cycle threshold) was calculated using MxPro software (Stratagene). For each gene, the amplification efficiency of the primer set was determined by performing quantitative PCR using a dilution series of the cDNA as a template, as previously described (Nyegaard et al. 2010).

### E_2_ enzyme-linked immunosorbent assay (ELISA)

We determined E_2_ in conditioned media using the ELISA kit from Bio-quant (San Diego, CA, USA) following the manufacturer’s instructions. Briefly, cells were treated with FSH and/or BPA in the presence of 4-androstene-3,17-dione; 48 hr later medium was collected, properly diluted, and stored at −80°C or immediately analyzed. Standards and positive controls (provided in the kit) or samples were placed into the wells of a microplate, along with the E_2_–horseradish peroxidase conjugate and rabbit anti-E_2_ reagent, and incubated for 2 hr at room temperature. Afterward, samples were decanted and wells were washed extensively. Finally, a stabilized chromogen was added, and the colorimetric reaction was developed within 30 min, after which “stop solution” was added and the absorbance was measured on a spectrophotometer at 450 nm. Additionally, remaining cells were homogenized in protein lysis buffer [10 mM Tris-HCl (pH 7.4), 100 mM NaCl, 1 mM EDTA, 1 mM EGTA, 1 mM sodium fluoride, 20 mM sodium diphosphate, 2 mM sodium orthovanadate, 1% Triton X-100, 10% glycerol, 0.1% sodium dodecyl sulfate (SDS), 0.5% deoxycholate], and the protein concentration in each well was measured. Final data are plotted as picograms of synthesized E_2_ per microgram of total protein. Cross-reactivity with estrone and estriol approximated 2.1% and 1.5%, respectively (the percentage indicates cross-reactivity at 50% displacement compared with E_2_).

### Western blot analysis

After adding treatments, we cultured cells for various periods of time; subsequently, media were removed and cells were washed with ice-cold PBS. Cytosolic fractions were prepared to evaluate aromatase levels, and nuclear fractions were used to assess the expression of PPARγ protein. Cells were lysed in “lysis buffer” (Pierce, Rockford, IL, USA), and proteins were extracted according to the manufacturer’s instructions. Briefly, cells were homogenized in lysis buffer I (cytosolic) and kept on ice for 10 min, after which suspensions were centrifuged at 16,000 × *g* for 5 min and supernatants (cytosolic fraction) were collected and stored at −80°C until analysis. The remaining pellets were resuspended in lysis buffer II (nuclear) and vigorously vortexed for 40 min at 4°C. The nuclear suspensions were centrifuged at 16,000 × *g* for 10 min, and supernatants (nuclear fraction) were collected and stored at −80°C until analysis. Shortly before Western blotting was performed, protein extracts were quantified using the BCA (bicinchoninic acid) Protein Assay Kit (Pierce). SDS–polyacrylamide gel electrophoresis was run on precasted, 4–20% Tris-HCl minigels for 1.5 hr at 120 V, and proteins were subsequently transferred onto polyvinylidene difluoride (PVDF) membranes in transfer buffer (25 mM Tris, 192 mM glycine, 20% vol/vol methanol, pH 8.3) for 1 hr at 50 V. Membranes were blocked with 5% milk/Tris-buffered saline–Tween 20, -T, and antibody incubation was performed overnight at 4°C. We used the following antibodies: aromatase (MCA2077S; Serotec, Raleigh, NC, USA), and actin (sc1616), PPARγ (sc7273), and lamin A/C (sc20681; all from Santa Cruz Biotechnology, Santa Cruz, CA, USA). To analyze loading accuracy, PVDF membranes were stripped in Western Blot Stripping Buffer (Pierce). Western blots were visualized using SuperSignal West Femto Trial kit (Pierce) or Lumigen PS-3 detection reagent (GE Healthcare, Pittsburgh, PA, USA), according to the manufacturer’s instructions. Developed immunoblots were exposed to high-performance chemiluminescence film (GE Healthcare).

### Plasmids and cell transfection

We obtained expression and control empty pBabe-bleo plasmids from R. Kahn (Joslin Diabetes Center, Boston, MA, USA) and R. Weinberg (Massachusetts Institute of Technology, Cambridge, MA, USA), respectively. Cells were seeded in 12-well plates in DMEM/F12 medium and incubated for 2 hr at 37°C. Meanwhile, 1 μL FUGENE 6 transfection reagent (Roche, Indianapolis, IN, USA) and 0.25 μg plasmid DNA per well were combined in 100 μL OPTI-MEM medium and allowed to incubate for 20 min at room temperature. Subsequently, the complexes were added to cells and incubated for another 2 hr at 37°C before treatments were added. Empty pBabe-bleo vector was added to keep the amount of transfected DNA constant in the PPARγ dose–response experiments.

### Bromodeoxyuridine (BrdU) ELISA

BrdU incorporation ELISA (Roche) was carried out according to the manufacturer’s instructions. Briefly, cells were seeded in 96-well plates and treated with tested additives. BrdU labeling solution (10 μL) was added to each 100 μL of culture medium for the last 12 hr of culture. Afterward, culture medium was decanted, and cells were fixed with 200 μL FixDenat solution (Roche) for 30 min at room temperature. Subsequently anti-BrdU working solution was added (100 μL/well) and incubated at room temperature for another 1.5 hr; after extensive washes, the colorimetric reaction was developed by adding the substrate solution. After 30 min the reaction was stopped by adding 1 M sulfuric acid, and the absorbance was measured at 450 nm. Results are presented as fold decrease and plotted as percentage of control.

### MTS proliferation assay

We performed the MTS cell proliferation assay (Promega), which is typically used to determine the number of viable cells in proliferation or cytotoxicity assays, according to the manufacturer’s instructions. Briefly, 20 μL of staining solution was added to cells at the end of respective treatments, and after 1–4 hr of incubation, MTS tetrazolium compound [3-(4,5-dimethylthiazol-2-yl)-5-(3-carboxymethoxyphenyl)-2-(4-sulfophenyl)-2H-tetrazolium, inner salt] was bioreduced by cells into a color formazan product, soluble in culture medium. The absorbance was measured at 490 nm, and results are presented in the same fashion as for the BrdU incorporation experiments.

### Low-molecular-weight DNA apoptotic ladder assay

We performed the low-molecular-weight DNA apoptotic assay (Roche) according to the manufacturer’s instructions. Briefly, at the conclusion of the cell culture studies, we added 200 μL/well of binding/lysis buffer to the cells and incubated them for 15 min at room temperature. DNA was precipitated by adding isopropanol, followed by vigorous shaking. Afterward, samples were run through DNA binding columns and spun gently. DNA was then washed twice with ethanol, eluted from columns by adding 200 μL water, followed by centrifugation. The final product was mixed with DNA loading buffer and subjected to 1% agarose electrophoresis for 2 hr at 75 V. Gels were then analyzed on the Bio-Rad Gel Doc ultraviolet camera system (Bio-Rad, Hercules, CA, USA). We purchased 20× TAE DNA electrophoresis buffer from Qiagen (Valencia, CA, USA); agarose and ethidium bromide were from Sigma-Aldrich, and 10× BlueJuice DNA gel loading buffer was purchased from Invitrogen.

### Statistical analysis

We performed statistical analysis using Prism software (GraphPad Software, Inc., San Diego, CA, USA). One-way analysis of variance (ANOVA), followed by the Tukey test, was used for all analyses.

## Results

### BPA reduces FSH-induced IGF-1 and aromatase expression, and E_2_ synthesis

**Figure 1:**
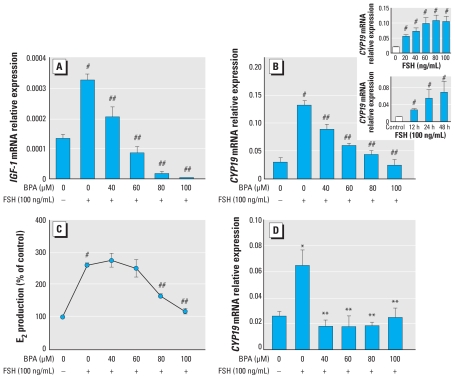
Effects of BPA on IGF-1, aromatase (CYP19), and E_2_ production by KGN cells and aromatase expression in human luteinized granulosa cells. (*A*) Dose–response inhibitory effects of BPA on relative expression of FSH-stimulated *IGF*-*1* mRNA after 48 hr of treatment. (*B*) Dose–response inhibitory effects of BPA on expression of FSH-stimulated aromatase (*CYP19*) mRNA after 48 hr of treatment. The insets represent dose–response (top) and time-course (bottom) experiments indicating the highest and most optimal response of KGN cells in terms of *CYP19* expression at 48 hr with 100 ng/mL FSH. (*C*) Effects of BPA on FSH-induced E_2_ secretion by KGN cells. Prominent reduction of E_2_ synthesis is noted at 80 and 100 μM BPA; Results were derived as pg E_2_/μg protein and are expressed as percentage of control. (*D*) Dose–response inhibitory effects of BPA on FSH-stimulated aromatase mRNA expression in human luteinized granulosa cells after 48 hr treatment. For all experiments, *n* = 5. *p < 0.05 versus control; **p < 0.05 versus FSH; ^#^*p* < 0.001 versus control; and ^##^*p* < 0.001 versus FSH, all by ANOVA, Tukey.

KGN cells express a functional FSH receptor (Nishi et al. 2001). Using this cell line, we found that FSH-induced expression of IGF-1, a factor known to play a crucial role in granulosa proliferation and steroidogenesis, was dramatically reduced by administration of BPA in a dose-dependent fashion (Figure 1A). With regard to aromatase, another FSH-stimulated gene in granulosa cells, treatment with 40–100 ng/mL FSH for 48 hr produced an optimal response (4.5-fold increase of aromatase mRNA; Figure 1B, inset). BPA causes down-regulation of FSH-induced aromatase mRNA expression in a dose-dependent fashion, reducing its expression to the level of control at the highest (60–100 μM) concentrations (Figure 1B). Consistent with the aromatase expression pattern, FSH-induced E_2_ synthesis was significantly reduced by BPA to the level observed in untreated cells (Figure 1C). Additionally, a similar response was observed with human luteinized granulosa cells cultured in the presence of FSH with and without BPA (Figure 1D). However, a sharp decrease in FSH-induced aromatase after BPA treatment in these cells occurred when the lowest tested concentration (40 μM) of BPA was used. BPA had no effect alone on IGF-1, aromatase, or E_2_ production by KGN cells (data not shown).

### BPA affects expression of aromatase regulators

**Figure 2:**
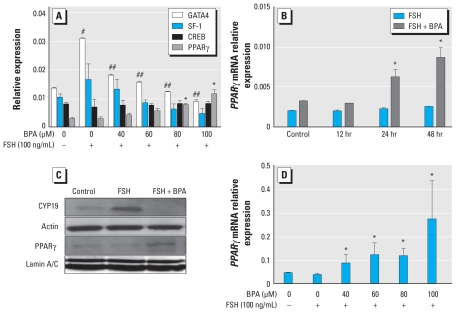
Effects of BPA on granulosa transcription factors. (*A*) Effects of FSH and FSH plus BPA (after 48 hr treatment) on mRNA expression of transcription factors known to be involved in FSH-induction of aromatase (GATA4, SF-1, CREB, and PPARγ). A post hoc linear trend test was used to analyze changes of SF-1 expression (*n* = 5 for each treatment group). (*B*) Stimulatory effects of BPA and FSH on relative expression of PPARγ in KGN cells over a 48-hr time course. Cells were treated with FSH (100 ng/mL) with or without BPA (100 μM) (*n* = 3 for each treatment group). (*C*) Effects of BPA on FSH-induced aromatase and PPARγ protein level in KGN cells, shown by representative Western blots for aromatase (CYP19) subsequently reprobed for total actin (cytoplasmic normalizer), PPARγ, and lamin A/C (nuclear normalizer). FSH and BPA were added to KGN cells at 100 ng/mL and 100 μM, respectively. (*D*) Dose–response stimulatory effects of BPA on expression of *PPAR*γ mRNA in human luteinized granulosa cells in the presence of FSH after 48 hr treatment (*n* = 3 for each treatment group). For *A, B*, and *D*, data are mean ± SEM. *p < 0.05 versus control; ^#^*p* < 0.001 versus control; and ^##^*p* < 0.001 versus FSH all by ANOVA, Tukey.

In an effort to determine what transcription factors may be affected by BPA inhibition of FSH-induced aromatase, we investigated the expression of select aromatase regulators: GATA4, SF-1, CREB (positive regulators of aromatase), and PPARγ (a negative regulator of aromatase), using KGN cells and the same experimental design described above. As shown in Figure 2A, the expression pattern of GATA4 is nearly identical to that of aromatase, being elevated by FSH and reduced by BPA in a dose-dependent fashion, with GATA4 expression reduced to the level of control at 80 and 100 μM concentrations of BPA. SF-1 followed a similar trend, although the changes showed a trend to significance (*p* < 0.054). However, a post hoc test for linear trend revealed statistical significance (*p* < 0.004). Interestingly, expression of CREB did not change, whereas expression of PPARγ, a factor that has been shown to participate in the inhibition of aromatase in KGN cells (Fan et al. 2005), increased with increasing concentrations of BPA. To determine if *PPAR*γ mRNA accumulation was due to FSH or FSH plus BPA (because these two additives were always administered together), we conducted a time-course experiment (Figure 2B). PPARγ expression was significantly elevated by 24 hr and sustained through 48 hr, and this was observed only in cultures in which FSH plus BPA were added concomitantly. Furthermore, BPA increased nuclear accumulation of PPARγ protein and also reduced FSH-induced aromatase protein levels in KGN cells, which confirmed the PCR results (Figure 2C).

We further investigated the effects of BPA on the expression of PPARγ in human luteinized granulosa cells (Figure 2D) and observed that BPA increased *PPAR*γ mRNA by 7-fold at the highest concentration (100 μM). The response of human luteinized granulosa cells to BPA, in terms of PPARγ expression, was greater than that of KGN cells at low concentrations (40 mM). We observed the same trend with aromatase, suggesting that the human luteinizing granulosa cells are more sensitive to this endocrine disruptor in lower concentrations, compared with the KGN cell line.

### Overexpression of PPARγ mimics BPA action

**Figure 3:**
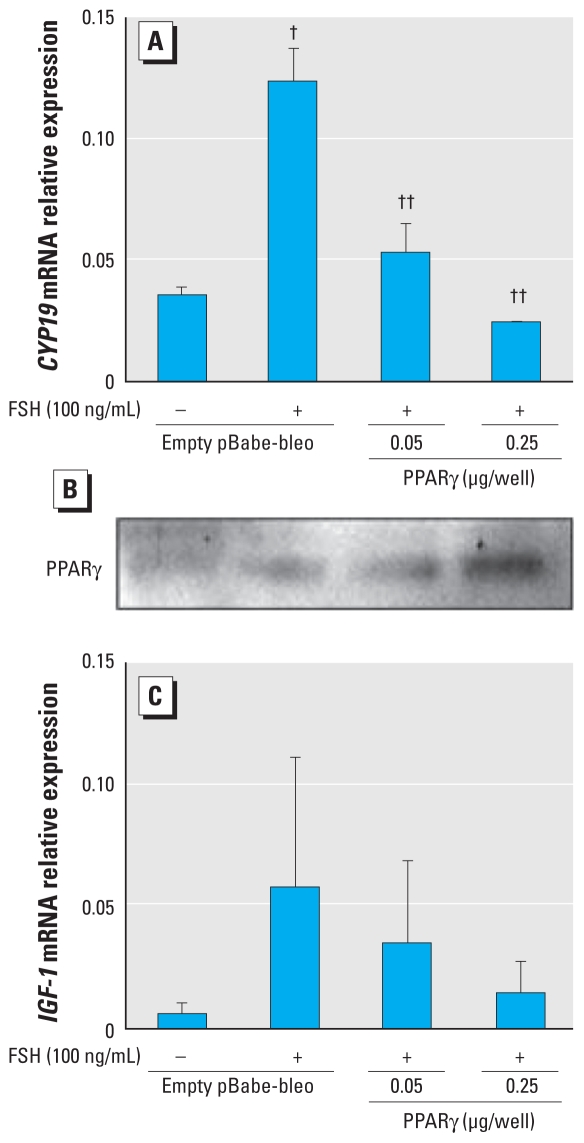
Overexpression of PPARγ mimics the effects of BPA on FSH-stimulated aromatase and IGF-1 expression. KGN granulosa cells were transfected with increasing amounts of the pBabe-bleo human PPARγ expression vector [the total amount of DNA was kept constant in all samples (0.25 μg/well) by adding empty plasmid (pBabe-bleo)]. Cells were treated simultaneously with FSH (100 ng/mL) to show effects of PPARγ expression on FSH-stimulated aromatase (*A*) and IGF-1 (*C*). A validating PPARγ immunoblot (*B*) was performed to evaluate the level of expressed PPARγ protein in this experiment. For *A* and *C*, *n* = 5. ^†^*p* < 0.001 versus control; and ^††^*p* < 0.01 versus FSH, both by ANOVA, Tukey.

To determine whether overexpression of PPARγ mimics BPA action, we transfected into KGN cells two doses of pBabe-bleo expression vector, either empty or carrying the PPARγ sequence, and treated the transfected cells with and without FSH. As shown in Figure 3A, FSH-induced aromatase is down-regulated with increasing concentrations of the PPARγ vector, and there was a similar trend of IGF-1 down-regulation (Figure 3C). A Western blot was performed to validate the presence of expressed PPARγ protein (Figure 3B). Together, these data confirm that increasing PPARγ expression mimics the action of BPA.

We also attempted RNA interference experiments to elucidate the affects of BPA on PPARγ expression. However, the combination of BPA and small interfering RNA transfection reagents appeared to be toxic to the KGN cells, even at low BPA concentrations (data not shown). Furthermore, addition of the PPARγ antagonist GW9626 to cells 1 hr before BPA treatment did not restore aromatase expression reduced by BPA (data not shown), likely because BPA-increased PPARγ expression peaks after 48 hr, when the action of the antagonist is no longer present. Thus, the overexpression data are the most robust evidence of PPARγ mimicking BPA action in granulosa cells.

### BPA inhibits proliferation of KGN granulosa cells

**Figure 4:**
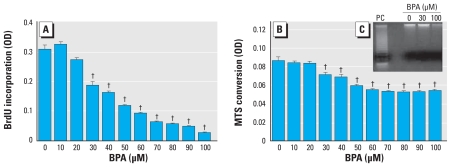
Effects of 48-hr BPA treatment on KGN cell survival, proliferation, and apoptosis. OD, optical density. (*A*) Effects of BPA on cell proliferation (BrdU incorporation). (*B*) Effects of BPA on cell viability; conversion of MTS tetrazolium to formazan corresponds directly with the metabolic status of living cells in culture. (*C*) DNA laddering of low-molecular-weight DNA; the positive control (PC) was 3 μM camptothecin. For each group, *n* = 8. ^†^*p* < 0.01 versus control by ANOVA, Tukey.

To determine if BPA affects granulosa cell proliferation, we conducted BrdU incorporation studies. As shown in Figure 4A, BPA inhibits KGN cell proliferation in a dose-dependent fashion, with maximum inhibition of 92 ± 2% at 100 μM BPA, compared with cells cultured with no additives. In a parallel assay evaluating cell viability, we observed a trend (Figure 4B) similar to that of proliferation (Figure 4A), although maximum reduction in cell viability (31 ± 3%) was achieved at 50 μM without further change at higher concentrations of BPA. To determine whether these changes were accompanied by cellular apoptosis, we conducted DNA laddering experiments. Low-molecular-weight DNA from cells treated with BPA at two different concentrations revealed no DNA fragmentation after BPA treatment, compared with cells treated with camptothecin, a known inducer of apoptosis (Figure 4C).

## Discussion

In the present study we found that the ubiquitous endocrine disruptor BPA impairs FSH-stimulated processes involved in ovarian steroidogenesis in human granulosa cells, including *IGF*-*1* mRNA expression, aromatase mRNA and protein, transcription factors involved in FSH-stimulated aromatase regulation, and E_2_ production. Furthermore, this inhibition occurs through accumulation of PPARγ, a member of the nuclear receptor superfamily of ligand-dependent transcription factors (Knouff and Auwerx 2004). Since its discovery, PPARγ activation has been mainly associated with processes such as increased fatty acid uptake and energy expenditure in liver, adipose tissue, and macrophages, as well as glucose uptake in muscle and adipocytes (Auwerx et al. 2003). However, PPARγ has also been shown to play important roles in regulating ovarian follicle development, ovulation, oocyte maturation, and maintenance of the corpus luteum by enhancing or inhibiting steroidogenesis, angiogenesis, tissue remodeling, the cell cycle, apoptosis, and lipid metabolism (Froment et al. 2006; Komar 2005). These processes are critical for normal ovarian function, so it is of significance that they are regulated by BPA, which alters expression of this nuclear receptor. It has been shown that PPARs are involved in the down-regulation of aromatase and estrogen synthesis as a consequence of phthalates, another class of ubiquitous environmental contaminant/endocrine disruptor (Lovekamp and Davis 2001; Lovekamp-Swan and Davis 2003; Lovekamp-Swan et al. 2003). Fan et al. (2005) demonstrated that activation of PPARγ down-regulates aromatase in human granulosa cells via inhibition of nuclear factor κB (NF-κB), a protein kinase C–dependent transcription factor (Diaz-Meco et al. 1993), which under physiologic conditions may compete with other nuclear receptors (e.g., SF-1, CREB, GATA4) to regulate aromatase, despite the absence of NF-κB’s binding domain in the aromatase proximal promoter. In the present study we provide evidence for a second potential mechanism responsible for aromatase inhibition, namely, via BPA-induced decrease of GATA4 and SF-1 transcription factors. Whether changes in expression of these regulators are directly involved in the BPA-mediated decrease in IGF-1 and CYP19 expression at the transcriptional level is of interest and worthy of further investigation.

An increasing body of evidence describing mechanisms involved in FSH-stimulated aromatase expression includes activation of the well-established array of cAMP-regulated transcription factors. It is also well documented that granulosa cell proliferation and steroidogenesis are a result not only of generation of cAMP upon ligand binding to the FSH receptor, but also of synergistic action of IGFs (Khamsi et al. 2001; Kwintkiewicz and Giudice 2009), locally synthesized or derived from the circulation. Interestingly, we also observed that BPA treatment dramatically reduced expression of IGF-1, thereby reducing IGF/FSH synergy, thus providing additional support for BPA as an endocrine disruptor. We observed that the reduction of IGF-1 by BPA may potentially be achieved through up-regulation of PPARγ because its overexpression revealed a trend to reduce IGF-1 expression, suggesting a key role of this nuclear receptor in BPA-mediated disruption of FSH-induced functions in granulosa cells. A recent study by Lecka-Czernik et al. (2007) demonstrated that rosiglitazone, a PPARγ agonist, suppresses components of the IGF regulatory system in different cell types, specifically blocking IGF-1 and IGF-2, as well as type 1 and type 2 IGF receptor expression, and that this is achieved by activation of PPARγ. This observation is in concordance with our findings and suggests that disruption of the ovarian IGF system may be one of several possible mechanisms by which the endocrine disruptor BPA exerts its effect on E_2_ biosynthesis in granulosa cells in the ovary.

Another important FSH/IGF-regulated function is follicular expansion driven by extensive proliferation of granulosa cells. Interestingly, in the present study our proliferation experiments demonstrated that granulosa cell BrdU incorporation was inhibited with increasing concentrations of BPA in the absence of cellular apoptosis. Furthermore, BPA exposure causes accumulation of PPARγ, with concomitant down-regulation of aromatase in human granulosa cells. Although precise mechanisms underlying PPARγ regulation by BPA and down-regulation of FSH-induced aromatase by overexpression of PPARγ in the presence of BPA in human granulosa cells are uncertain, some key observations in other systems may give insight into these regulatory processes. For example, BPA promotes rapid induction of nongenomic systems such as the mitogen-activated protein kinase (MAPK) pathway (Zsarnovszky et al. 2005), and through activation of this signaling cascade, BPA acts to modulate PPARγ activity. Activation of PPARγ may be achieved through ligand binding or by its phosphorylation through stimulation of signal transduction pathways such as MAPK (Chen et al. 2003). Although it seems that BPA is not a likely candidate to be a PPARγ ligand, it is known to activate—at nanomolar range concentrations—components of the MAPK pathway, such as phosphorylation of extracellular regulated protein kinase (Erk) 1/2 (Song et al. 2002; Zsarnovszky et al. 2005). Thus, elevated expression of PPARγ by BPA may be potentially important for its final activation, which occurs independently of the increase of its mRNA and protein level. Previous studies demonstrate that troglitazone-triggered activation of PPARγ leads to inhibition of aromatase via NF-κB (Fan et al. 2005), which provides a rationale and evidence that activated PPARγ contributes to the down-regulation of aromatase. Also, activation of Erk1/2 leads to phosphorylation of PPARγ, which leads to its physical association with the p65 subunit of NF-κB (Chen et al. 2003), supporting NF-κB as a mediator of BPA-associated aromatase inhibition. Further studies are needed to elucidate the role of NF-κB and the MAPK pathway in BPA-mediated aromatase inhibition in human granulosa cells.

Of interest are the dramatic and somewhat surprising results of BPA inhibition of BrdU incorporation (Figure 4) in the KGN cell line, in the absence of cellular apoptosis and cell death. It would be anticipated that at higher doses the cell numbers would be lower than at lower doses and that this may affect the results. However, because the data have been analyzed and reported as relative expression to a normalizer (e.g., actin or total protein), it is unlikely that this inhibition of DNA synthesis would change the interpretation of our data.

BPA has been shown to act on cells to modulate the state of their DNA methylation, ultimately altering promoter activity of, for example, ERα in the rat brain (Monje et al. 2007) or by decreasing cytosine–guanine dinucleotide methylation, causing prenatal epigenetic reprogramming (Dolinoy et al. 2007). Whether such actions (and others) of BPA are operational in regulation of PPARγ in human granulosa cells is currently under investigation in our laboratories.

The full impact of BPA as an endocrine disruptor in human ovarian steroidogenesis during adult exposures to this compound and the potential effects on compromised fertility remain to be elucidated. However, a recent preliminary report demonstrated an inverse correlation between serum levels of BPA (maximum of 30 nM) and lower fertilization rates of oocytes derived from women undergoing *in vitro* fertilization (IVF) (Lamb et al. 2008). These data thus suggest that BPA, a ubiquitous endocrine disruptor, may affect human granulosa steroidogenic potential *in vivo* and female fertility. In the present study, the effects of BPA on FSH-stimulated processes occurred at relatively high concentrations, with human luteinized granulosa cells seemingly more sensitive than the KGN cell line. Although circulating levels of BPA are lower than those used in our experiments, it is unclear whether women attain such high levels of BPA in their serum or follicular fluid by exposure to this chemical; such elevation of BPA would likely be transient after a specific exposure because BPA is not a persistent chemical. However, repeated exposures to a multitude of consumer products containing this endocrine disruptor may result in high concentrations. Given that high doses of BPA have such a profound effect on granulosa steroidogenesis, disruption of ovarian steroidogenesis leading to decreased reproductive potential is a real possibility. Minimizing such exposures to maximize reproductive potential, although not proven, is prudent in view of our data.
